# A giant lumbar chordoma: A case report

**DOI:** 10.1097/MD.0000000000011128

**Published:** 2018-06-22

**Authors:** Yibiao Zhou, Bolin Hu, Zhiwei Wu, Hanxiong Cheng, Min Dai, Bin Zhang

**Affiliations:** Department of Orthopaedics, The First Affiliated Hospital of Nanchang University, Nanchang Shi, Jiangxi Sheng, China.

**Keywords:** chordoma, giant, lumbar, surgery resection

## Abstract

**Rationale:**

Chordomas are malignant neoplasms derived from incomplete regression of notochordal tissue along the craniococcygeal axis.It is rare for Chordoma arising from the lumbar spine and the traditional long-term prognosis is typically poor.

**Patient concerns:**

The persistent pain in the left side of the waist about 2 years.

**Diagnoses:**

Chordoma.

**Interventions:**

The patient was treated with surgical resection of the total tumor, followed by the spinal internal fixation of L1 to L2 with pedicle screws.

**Outcomes:**

After 5 month follow-up,we find the recurrence in the original lesion.At the 15 month follow-up,the patient was dead after a lot of times revisit by various doctor.

**Lessons:**

So It is suggest that the diagnosis should be carried out accurately at the early stage, the lesions and source of lesions should be cut away as broadly as possible, also the radiation and chemotherapy should be carried out after the operation as necessary.

## 1 Introduction

Chordoma is a rare, locally invasive, and slow-growing malignant tumor that occurs with an total incidence of less than 1 in 1,000,000 people.^[18]^ A lot of studies suggest that the chordoma arise from remnants (rests) of the embryonic notochordal cells. The development of chordoma in men is more than in women (ratio 2: 1), and lesions typically arise in the sacrococcygeal area (29–57%), clivus area (27–35%), and vertebrae (10–33%).^[18]^ In the spinal columns, the tumor is located in the cervical spine (15–52%), lumbar spine (33–35%), and thoracic spine (14–17%).^[15]^ Histological examination described that these tumor cells often are separated by variable amounts of intercellular mucin and contain numerous intracytoplasmic vacuoles, accounting for 1% to 4% of all bone neoplasms.^[1]^

Here, we herein review the case of a 72-years-old patient with L1-2 vertebral huge chordoma who showed neurological symptoms because of tumor invasion into the neural foramen and canal, compressing the spinal cord and nerve root.

## 2 Case report

The patient was a 72-year-old man who was followed up for persistent pain in the left side of the waist for 2 years, radiating to the left hip. The history of schistosomiasis was 40 years, and with hypertension 1 year. On physical examination, he had pain on movement of the spine with bilateral Grade IV motor strength in both lower extremities. There was not numbness in both feet and ankles with normal ankle reflexes bilaterally. There was a normal knee reflex bilaterally. There was no obvious bulge in the bilateral kidney region, and the left lateral kidney region knocks out to be positive for pain. The neurological symptoms were ascribed to the compression of the spinal cord and nerve roots by an expanding mass partly protruding into the vertebral canal.

**Figure 1:**
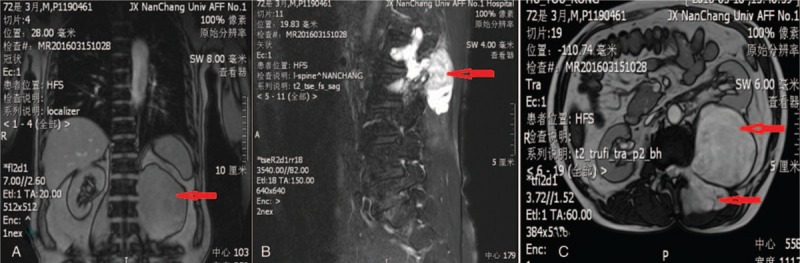
(A, B) Coronal and axial MRI revealed a large inhomogeneous mass causing the compression of the left kidney. (C) Sagittal MRI showed that the vertebral laminae of L2 was eroded and the left L2 intervertebral foramen was enlarged.

**Figure 2:**
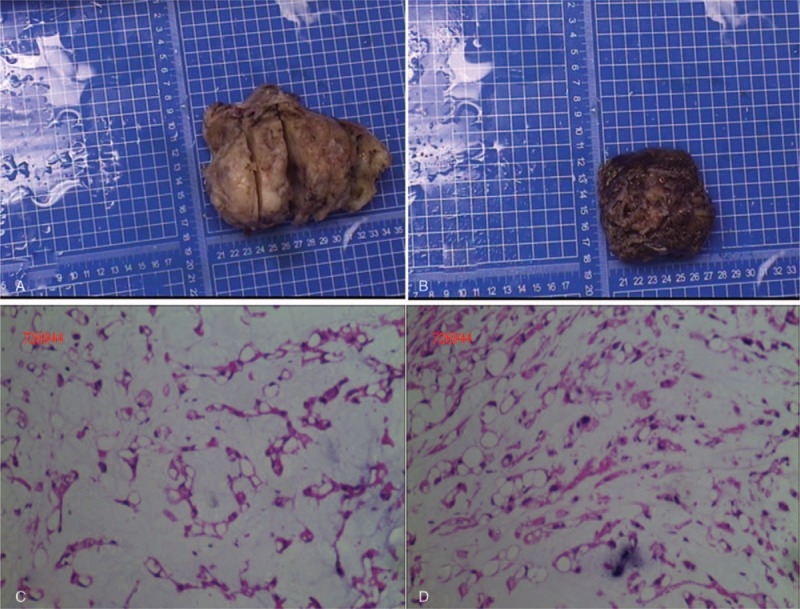
CT angiography did not suggest the blood supply of the mass.

Magnetic resonance imaging (MRI) was performed that the left side of the vertebral body and the left peritoneum showed an elliptical abnormal signal shadow, which showed a dumbbell growth. The MRI scan showed an area of increased signal intensity on T2-weighted images in the T12-L4 paravertebral region. At discovery, on the left side of the L1 vertebral body, there was a slightly longer T1/T2 signal, with a size of 8.6 × 8.4 × 12.4 cm (Fig. 1). The boundary was clear, and the enhanced scan showed an uneven separation. The lesion protruding into the erector spinae muscles and behind the retroperitoneum are connected to the lesion in the vertebral canal through the intervertebral foramen, and the spinal cord was compressed and the boundary was clear. And the computed tomography (CT) showed that bone destruction can be seen on left lateral transverse process of L2, and the left kidney was significantly compressed (Fig. 2).

**Figure 3:**
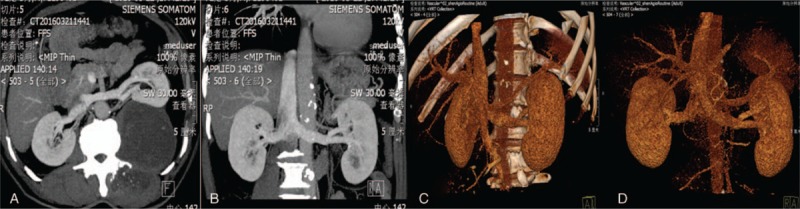
(A) and (B) were the mass removed from the lesions during the operation. Histological sections of the mass displayed tumor tissue in a myxoid background and cords and lobules of vacuolated physaliphorous cells with abundant cytoplasm and a large amount of mucus (C, D). The nucleus was round or oval without definite mitosis (original magnification, 100).

The patient was treated with surgical resection of the total tumor, followed by the spinal internal fixation of L1 to L2 with pedicle screws. A posterior approach was selected for decompression. Intraoperatively, 2 lumps measuring 10 × 9 × 8 cm and 8 × 6 × 3 cm were visible in the focal region. They were well circumscribed and completely resected (Fig. 3). Total laminectomy was performed for L1 and L2. On histopathological examination, the gross specimen consisted of ragged pieces of pale-tan, slightly gelatinous material admixed with blood clot. Microscopic examination of the formalin fixed paraffin-embedded specimen showed the presence of a lobulated neoplasm in a prominent extracellular myxoid matrix. The neoplastic cells were composed of syncytial cords of “physaliphorous cells” that contained abundant pale septated and vacuolated clear to bubbly cytoplasm with mild nuclear pleomorphism. Overt mitosis was not identified. Immunohistochemical examination showed that the lesional cells were positive to CK(+++), S100(++), CD65(+), neuron-specific enolase(++), epithelial membrane antigen (+++), and vimentin(+++). The coexpression of epithelial and mesenchymal immunohistochemical markers supported the notochordal lineage of the lesional cells. Ki67 was 3%+ in the lesional cells. The above histomorphological features in conjunction with the immune-phenotype observed confirmed the diagnosis of chordoma.

**Figure 4:**
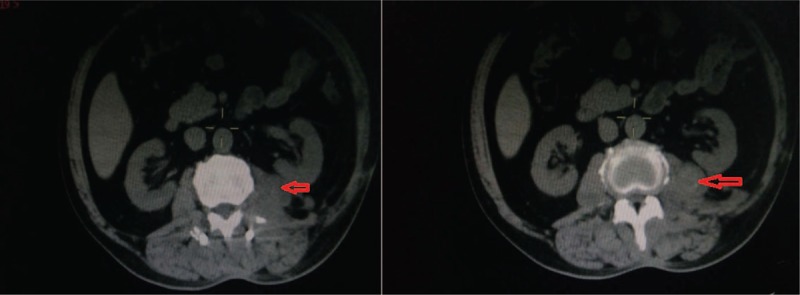
After 5 months, CT examination showed that the left lesion of the vertebral body appeared again (as arrow heads).

No heavy ion radiotherapy was performed because the tumor massively invaded into spinal canal and foramen, compressing the spinal cord and nerve root directly. After 5-month follow-up, we find the recurrence in the original lesion through the post-operative CT (Fig. 4). In consideration of the huge and complex recurrence, we did not have second operation. At the 15-month follow-up, the patient was dead after a lot of times revisit by various doctors.

## 3 Discussion

Chordoma is considered to be the tumor, which is derived from notochordal remnants.^[2,3,5,7,9,14,19,23]^ The incidence of chordomas is 5/10,000,000, which are rare, low-grade tumors.^[3,5,8]^ Comparing with women, chordoma is far more commonly seen in men.^[5,8,23,24]^ Chordoma is the most common primary malignant bone tumor throughout the spine, as is the sacrum.^[5]^ In the vertebral chordomas, the lumbar spine was about 40%, and the cervical spine was most common (nearly 50%).^[24]^ But in some studies, lumbar chordomas are more rare, accounting for approximately 6% in all spine chordomas.^[14]^ As seen in some reports, the patients diagnosed as chordoma were younger and younger, contrast to the age 60 years reported before.^[10,24]^

The clinical manifestations of paravertebral chordomas are mainly due to the compression of adjacent tissues caused by tumors, especially to the spinal cord and nerve roots, and even to the chest or abdominal organs. Low back pain is a common symptom of thoracolumbar spinal chordoma. Patients with saddle area chordoma are more prone to dizziness and other symptoms. The symptoms and imaging features of lumbar spine chordoma are not specific, and the pathological findings are required to establish the diagnosis. Therefore, it is easy to be confused with some rare tumors such as giant cell tumor and neurilemmoma before surgery. In this case, the patient's tumor crosses the intervertebral foramen. Therefore, the neurilemmoma are highly suspected before surgery.^[12]^

The case is reported in the position of L1, 2 segments, and as previously reported in literature, lumbar spinal cord tumors are very rare, what is more, we can observe that the volume of the chordoma is very huge, developing both directions to the posterior muscle and the left posterior peritoneal direction, the growth range from T12-L4, presenting a dumb-bell appearance and expanding through the enlarged intervertebral foramen. And bone destruction can be seen on left lateral transverse process of L2. On the basis of experience, the patient must have a very long tumor growth history. Because the preoperative diagnosis of chordomas is still challenging, delayed diagnosis occurs very frequently. CT and MRI are currently used in the diagnosis of chordomas. MRI signals of the mass are commonly isointense or hypointense on T1-weighted images and hyperintense on T2-weighted images. However, several other diagnoses including neurogenic tumors and lymphoma should also be considered. In our case, the initial diagnosis was a neurilemoma. Not just neurilemmoma, the patient's symptoms are also similar to the clinical manifestation of the lumbar disc herniation, so when the similar cases are appearing to us, we should have to give high vigilance in the chordoma, especially paying more attention on the imageological diagnosis and postoperative pathology.

The rates of local recurrence and metastasis depend on the quality of the initial surgery, pathological features, and age of the patient. In the present case, gross total removal of the chordoma was achieved followed by the spinal internal fixation of L1 to L2 with pedicle screws. This resulted in successful spinal stabilization without neurological deficiency, and in the initial stage, the patient was able to obtain good performance in activities of daily living without lower and upper limb paralysis, that is, good functional prognosis. But after 5 months, the recurrence was found at the patient's back with a raised mass. And then the patient was dead at 15 months after the surgery. On the basis of available information, the lesions cannot be completely removed. In view of that requirement of the patient's neural function reserve in the early stage, extensive excision of the adjacent tissues, such as the intervertebral body, nerve root, etc, has not been carry out extensively, as the function of the patient can be restored, and the patient's old age is taken into account, thus the therapeutic means for subsequent radiation therapy is abandoned. So, it is suggested that the diagnosis should be carried out accurately at the early stage, the lesions and source of lesions should be cut away as broadly as possible, also the radiation and chemotherapy should be carried out after the operation as necessary.

More recent studies of chordomas have been based on molecular biology to increase the survival rate. Osaka et al^[10]^ report that miR-155 expression will lead to the poor prognosis in the chordoma patients. This idea has been verified in their cell experiments. However, when the subject was changed to miR-1, Duan et al^[16]^ observed the opposite result.^[17]^ Zhang et al^[4]^ find that some miRNAs have a negative correlation with some molecules, such as miR-34a/MET and miR-608/EGFR in chordoma cells. Feng et al^[6]^ suggest that targeting PD-L1 may be a novel immunotherapeutic strategy for chordoma clinical trials. The findings of Hu et al^[20,21]^ highlight that fibroblast growth factor receptor (FGFR)/ methyl ethyl ketone (MEK)/ extracellular signal regulated kinase (ERK)/brachyury pathway coordinately regulates chordoma cell growth and survival and may represent a novel chemotherapeutic target for chordoma. Jäger et al^[22]^ suggest that the differential expression of *HOX* genes in chordomas of the clivus and sacrum suggests an oncofetal mechanism in gene regulation linked to the anatomic site. In the study from Chen et al,^[13]^ human (hsa-) miR-185-5p was identified as a crucial miRNA in chordoma development via the Wnt signaling pathway. Wen et al^[11]^ indicate that Sam68 is an independent prognostic factor for the local recurrence-free survival of sacral chordomas [hazard ratio = 5.929, 95% confidence interval (95% CI): 1.092–32.188, *P* = .039]. Thus, inhibition of certain molecules and their respective pathways could be a potential therapy for chordoma.

## 4 Conclusion

Chordomas are uncommon neoplasms that arise from the embryonic remnants of the notochord. It is very helpful for us to establish the diagnosis through CT and MRI, but histopathological examination still was the gold standard of accurate diagnosis on tumors, which can provide more useful characteristic indicators of chordoma. Surgical resection of the total mass remains the main method of treatment, and in many cases, postoperative adjuvant radiotherapy is also necessary. Nowadays, more and more molecular biological studies are being performed to explore the pathogenesis of chordoma, and therefore targeted therapy may be an option for related patients in the future. Furthermore, in order to change the status of the poor prognosis for chordomas, a postoperative follow-up protocol should be established and executed; more studies should be performed to fully expound the characteristic of chordoma.

## Author contributions

ZYB conceived and conducted the experiments and prepared the manuscript. HBL contributed to sort and analyze the data. WZW, CHX helped to perform the analysis with constructive discussions. ZB and DM contributed to the conception of the study and approved the final manuscript. All authors read and approved the final manuscript.

**Conceptualization:** Bin Zhang, Min Dai.

**Data curation:** Yibiao Zhou, Bolin Hu, Zhiwei Wu.

**Formal analysis:** Yibiao Zhou, Bolin Hu, Bin Zhang.

**Funding acquisition:** Yibiao Zhou, Min Dai.

**Investigation:** Yibiao Zhou, Bolin Hu, Bin Zhang.

**Methodology:** Bolin Hu, Zhiwei Wu, Hanxiong Cheng, Bin Zhang.

**Project administration:** Hanxiong Cheng.

**Resources:** Hanxiong Cheng.

**Software:** Zhiwei Wu.

**Supervision:** Hanxiong Cheng.

**Validation:** Zhiwei Wu.

**Visualization:** Hanxiong Cheng.

**Writing – original draft:** Yibiao Zhou.

**Writing – review & editing:** Bin Zhang, Min Dai.
